# Detection of *Babesia conradae* in Coyotes (*Canis latrans*) and Coyote-Hunting Greyhound Dogs (*Canis familiaris*)

**DOI:** 10.3390/pathogens12040528

**Published:** 2023-03-29

**Authors:** Haley Furman, Ruth C. Scimeca

**Affiliations:** Department of Veterinary Pathobiology, College of Veterinary Medicine, Oklahoma State University, Stillwater, OK 74078, USA

**Keywords:** *Babesia conradae*, piroplasms, greyhounds, coyotes, transmission

## Abstract

*Babesia conradae* is a small piroplasm previously detected in coyote-hunting Greyhound dogs in California and Oklahoma. In dogs, *B. conradae* causes clinical signs similar to other tick-borne illnesses, and if not treated it can lead to acute kidney injury and other life-threating complications. To date, the life cycle of this apicomplexan parasite has not been fully described, but suggestions of direct contact or tick transmission have been proposed. The purpose of this study was to test coyote tissue samples from coyotes hunted by Greyhound dogs with a history of *B. conradae* infection to determine if this parasite is present in the coyote population in Northwestern Oklahoma. The analyzed tissue samples included liver, lung and tongue samples collected by hunters. DNA was isolated from these tissues and assessed by RT-PCR of the 18S rRNA and PCR of the COX1 genes for *B. conradae*. A total of 66 dogs and 38 coyotes were tested, and the results demonstrated the presence of *B. conradae* DNA in 21 dogs (31.8%) and 4 coyotes (10.5%). These results indicate that *B. conradae* is present in the dog and coyote population from the same area and that direct contact with coyotes may increase the risk of infection in dogs. Further studies are required to test possible modes of transmission, including direct bite, tick or vertical transmission.

## 1. Introduction

*Babesia conradae* (Kjemtrum et al., 2006) is a parasite within the phylum Apicomplexa known to infect dogs (*Canis familiaris* Linneaus, 1758). It was first reported as a novel species in California by Kjemtrum in 2006 [1], based on phylogenetic analysis and on a previous study related to piroplasms diversity [2]. *B. conradae* has been classified as *Babesia sensu lato*, and it has been included in the clade III according to the phylogenetic lineages of the 18S rRNA gene [3]. Among the conditions associated to *B. conradae* infection in dogs are hemolytic anemia, thrombocytopenia and splenomegaly, with a variable outcome if not treated [4,5]. Acute kidney injury and acute respiratory distress syndrome can, among other complications, occur in infected dogs [6]. The current recommended effective therapy is a combination of Atovaquone and Azithromycin [5]. Reported infected canines have been dogs from California and Oklahoma [1,5,6,7]. As for most *Babesia sensu lato*, the life cycle of *B. conradae* has not been described yet; however, because a majority of cases in dogs have been detected in Greyhound and Greyhound-mix coyote-hunting dogs, the possibility of a direct animal-to-animal transmission by bite wound exists. However, a few cases have also been reported from mixed-breed dogs, including a bull-terrier-type female dog and three littermates that were only 6 weeks old [5]. In addition to these cases, a report of *B. conradae* detection in two *Dermacentor albipictus* (Packard, 1869) nymphs collected from cats in the states of Minnesota and Colorado was published by Duncan in 2021 [8]. The authors indicated that this finding does not establish the transmission of *B. conradae* by *D. albipictus* but rather encourages further studies. The purpose of the present study was to evaluate the presence of *B. conradae* in coyote (*Canis latrans* Say, 1823) tissues hunted by Greyhound dogs in Northwestern Oklahoma and collect preliminary data for further studies. We initially tested the Greyhound hunting dogs and subsequently analyzed tissues from coyotes collected by Greyhound coyote-hunting dog owners from the same area where the dogs have been tested positive for *B. conradae.* Testing for the presence of this protozoan parasite in the coyote population will provide information related to the risk of infection among domestic dogs that are in close contact with coyotes.

## 2. Materials and Methods

### 2.1. Animals and Sample Collection

#### 2.1.1. Greyhound Blood Samples

A total of 66 Greyhound dogs from 9 kennels in Northwestern Oklahoma (coordinates 36.73° N 98.32° N), utilized for coyote hunting, were included in the analysis. With owner consent, whole blood was collected in EDTA tubes by venipuncture of the cephalic vein and transported with ice packs for DNA isolation during April 2021 to the parasitology research laboratory at Oklahoma State University, Stillwater, OK, USA.

#### 2.1.2. Coyote Tissue Samples

A total of 38 coyotes were sampled during the months of November 2021 to March 2022. The samples included 38 tongues, 25 lungs, and 21 liver tissues, with only 13 coyotes having all 3 tissues. Approximately 50 g of each tissue were collected by Greyhound-coyote-hunting owners in the area were *B. conradae* was previously detected in dogs, as described above. The tissues were placed in Ethanol 70%, submitted to the laboratory and stored at −20 °C until further processing.

### 2.2. Tick-Borne Pathogens-Antibody Detection in Dogs

At the time when blood was collected, all the dog owners were questioned about the history of tick-borne illness during the previous hunting season (October 2020 to March 2021). The reason to test for tick-borne pathogen antibodies was because some of the dog owners described several dogs with lethargy and fever during the winter months of 2020–2021, with 6 of the dogs being previously treated with Doxycycline during the month of November 2020. We utilized the commercially available VETSCAN Flex4 rapid test, catalog number 100-21-677 (Zoetis, Parsippany, NJ, USA). This immunochromatographic test uses lateral flow technology and detects antibodies to *Anaplasma phagocytophilum* (Foggie, 1949) Dumler et al., 2001 [9], *Anaplasma platys* Dumler et al., 2001 [9], *Borrelia burgdogferi* (Johnson et al., 1984) Adeolu and Gupta 2015 [10], *Ehrlichia canis* (Donatien and Lestoquard 1935) Moshkovski 1945 (Approved Lists 1980) emend. Dumler et al., 2001 [9], *Ehrlichia chaffeensis* Anderson et al., 1992 emend. Dumler et al., 2001 [9,11] and *Ehrlichia ewingii* Anderson et al., 1992 emend. Dumler et al., 2001 [9,11], and it also detects the presence of *Dirofilaria immitis* Leidy, 1856 antigen. The test was performed following the manufacturer’s instructions. Briefly, 1 drop of whole blood was added to each sample well (2 in total), followed by the addition of 3 drops of the buffer solution provided in the test. Both the sample and buffer solution were at room temperature before running the test. The colorimetric results on the VETSCAN Flex4 rapid test were read exactly 8 min after the addition of the buffer solution. The test was considered valid by the detection of the positive control line.

### 2.3. Peripheral Blood Smears in Dogs

Blood smears were stained with the modified Romanowski method: Dip Quick stain, catalog number J0322 (JorVet, Loveland, CO, USA), following the manufacturer’s instructions, and examined by light microscopy, using the Olympus BX43 microscope (Tokyo, Japan).

### 2.4. DNA Isolation and Purification

DNA was isolated from Greyhound dogs’ whole blood, using the Relia Prep Blood gDNA miniprep system (Promega, Madison, WI, USA), according to the manufacturer’s instructions, and stored at −20 °C. For the coyote DNA tissue extractions, the QuickDNA Miniprep Plus Kit (Zymo Research, Irvine, CA, USA) was used, followed by removal of PCR inhibitors with the OneStep PCR Inhibitor Removal Kit (Zymo Research, Irvine, CA, USA), both protocols following the manufacturer’s directions.

### 2.5. Babesia Conradae DNA Detection and Sequencing in Dogs and Coyotes

An RT-PCR using the LSU primers [9] that amplifies a region of the 18S ribosomal gene in *B. conradae* was performed. The RT-PCR was performed as previously described in [12]. A total of 12.5 µL iTaq Universal SYBR Green Supermix (BioRad, Hercules, CA, USA), 9.5 µL RNAse-free water, 2–5 µL DNA sample, and 0.5 µL of each primer were used for the RT-PCR reaction. Three previously sequenced *B. conradae* samples obtained from an earlier study [6] were used as positive controls. The DNA concentration and quality were assessed using the Nanodrop^TM^ 8000 Spectrophotometer, catalog number ND-8000-GL (Thermo Fisher Scientific, Carlsbad, CA, USA).

Additionally, to perform the sequence analysis, a PCR that amplifies a 490 bp fragment of the *B. conradae* COX1 gene was implemented using the primers designed for this study: SBcCOX1F: ACTGGATGGACTTTGTACCCT and SBcCOX1R: GCCCCCATACTAAACATCCA. The PCR conditions used were as follows: Pre-denaturation at 94 °C for 3 min, 35 cycles of denaturation at 94 °C for 30 s, an annealing temperature of 56 °C for 30 s and extension at 72 °C for 1 min, followed by a final extension of 72 °C for 5 min. The PCR products were separated in agarose 1.25% and observed by using the GelRed Nucleic Acid Stain (Biotum Fremont, CA, USA).

Amplicons were purified using the Wizard SV Gel and PCR Clean-Up System (Promega, MA, USA) according to the manufacturer’s instructions and submitted to Eurofins Genomics for sequencing (Louisville, KY, USA). Sequences were compared to the Nucleotide BLAST database (National Library of Medicine, Bethesda, MD, USA) and aligned using the Geneious Prime Software V.2023.0.4 (Boston, MA, USA).

## 3. Results

### 3.1. Tick-Borne Pathogens-Antibody Detection in Dogs

The results for the tick-borne pathogen antibodies in the Greyhound dogs are summarized in Table 1. We did not detect *Dirofilaria immitis* antigen on any of the dogs.

### 3.2. Peripheral Blood Smears in Dogs

No parasites or tick-borne pathogens were observed by light microscopy on any of the blood smears.

### 3.3. Babesia Conradae DNA Detection and Sequencing in Dogs and Coyotes

A total of 21 dogs (31.8%) tested positive for *B. conradae* using both the 18S rRNA RT-PCR and COX1 PCR during April 2021 (Table 1), with no mismatches found. Four of the dogs positive for *B. conradae* RT-PCR and PCRs were antibody-positive of *Anaplasma* spp., and two dogs positive for *B. conradae* were also antibody-positive for *Ehrlichia* spp. (Table 1).

Four of the 38 (10.5%) coyotes tested positive for *B. conradae* using the 18S rRNA RT-PCR and COX1 PCRs. *B. conradae* DNA was detected in four tongue samples, including one coyote for whom it was detected in both tongue and lung tissues.

**Figure 1:**
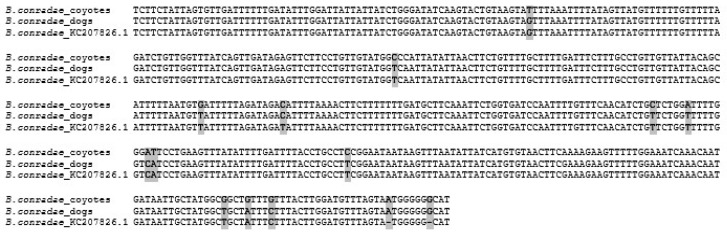
Base-pair difference of the COX1 gene region amplified in this study from dogs and coyotes compared to the sequence available in GenBank.

Sequences from dogs and coyotes were correspondingly similar and were deposited in the GenBank under the accession numbers OQ362168 and OQ362169, respectively. The analysis of the COX1 gene revealed a 99.9% similarity between *B. conradae* from dogs and coyotes. The sequences obtained in this study were 99.6% similar to the COX1 sequences previously published in GenBank (Figure 1).

## 4. Discussion

The life cycle of *B. conradae* has not been described yet; however, a hypothesis of transmission from ticks or via bite wounds has been proposed [6,7]. In Oklahoma, the time of the year when coyote hunters use their dogs to hunt ranges between late October and the beginning of March. Because of this, we first tested the Greyhound dogs used for coyote hunting at the end of the coyote-hunting season, at the beginning of April 2021. Following confirmation of *B. conradae* presence in hunting dogs, we analyzed tissues from coyotes collected by hunters during the upcoming hunting season, between October 2021 and March 2022; samples were obtained from the same area where the dogs were first detected as being *B. conradae*-positive and where the coyotes were hunted by the same dog population. Even though a previous report of *B. conradae* in Greyhounds in the state of Oklahoma has been published [6], to the authors’ knowledge, this is the first report describing the presence of *B. conradae* in coyotes in Oklahoma. A previous study by Javeed et al., 2022 [13] described an analysis of coyote tissues from Southern California, with a prevalence of 4.7%. In our study, the prevalence in the coyote population from Northwestern Oklahoma was 10.5%. This result could be explained by the fact that in the area in which we tested coyotes, *B. conradae* had been previously detected in the Greyhound population, while in the study from California the samples were conveniently collected by the hunters in an extended geographical area. A total of 38 coyotes were evaluated in this study. Twenty-five of the coyotes had two tissue types, tongue and lung, and thirteen coyotes had three tissue types, including the liver. Unfortunately, other tissues, such as the kidney or spleen, were not possible to obtain, and in most of the cases the tongue was the most accessible tissue to collect. DNA testing from the spleen is often used when whole blood is not possible to collect [6]. The reason why *B. conradae* DNA was detected mostly in tongue tissues is still unknown, but it could be due to vascularization or distribution of the parasite in the host. 

So far, to our knowledge, no information regarding *B. conradae* infection in coyotes and related clinical signs has been published. The information obtained in this study, along with the work by Javeed et al. [13], indicates that coyotes are being infected with *B. conradae*, but we do not know if, like in Greyhound dogs, they are also capable of developing clinical signs, such as in the case of infection with *B. gibsoni* [14]. Infection with *B. gibsoni* in coyotes was demonstrated by an experimental study using captive raised coyotes, where they developed clinical signs similar to the ones observed in domestic dogs [14]. Previous knowledge regarding the modes of transmission of *B. gibsoni* could help in discovering possible routes of infection for *B. conradae*, such as blood transfusion, tick bite, vertical transmission, or animal-to-animal bite wounds [15,16,17,18]. Further steps that can help us acquire more information related to the life cycle of *B. conradae* involve evaluating the presence of the parasite in ticks in the area or developing an experimental model of infection in domestic dogs. Investigations on whether Greyhound dogs and Greyhound mixes [7] are more susceptible to developing clinical signs when infected with *B. conradae* compared to other breeds should also be further pursued; therefore, the experimental model of infection and the testing of more dogs from different breeds could provide information to answer these questions.

In conclusion, the findings of *B. conradae* DNA in tongue and lung tissues from coyotes in Northwestern Oklahoma indicate that the pathogen is circulating in the coyote population and that dogs that are in direct contact with the coyote population could be at a greater risk of becoming infected with this protozoan parasite. Although to date most of the reports related to *B. conradae* infection in dogs have been associated with Greyhound dogs and Greyhound dog mixes used as aids during coyote-hunting activities, transmission via tick bites is another possibility. Since *B. conradae* DNA was detected in both dogs and coyotes from the same area, ticks in the area could be transmitting the pathogen to both canids. Further studies should be pursued to identify other possible modes of transmission, to assess the risk for the pet dog population and to describe the life cycle of this protozoan parasite.

## Figures and Tables

**Table 1:** VETSCAN Flex4 rapid test and *B. conradae* DNA detection in Greyhound dogs.

Kennel Number	Total of Dogs	*Anaplasma* spp. ^1^	*Borrelia* spp. ^1^	*Ehrlichia* spp. ^1^	*Babesia conradae* ^2^
1	12	1 *	0	0	6 *
2	4	0	0	0	2
3	11	0	0	2 *	1 *
4	7	0	0	0	0
5	11	1 *	0	0	2 *
6	9	3 *	0	2	3 *
7	4	1 **	0	1 **	2 **
8	4	0	0	0	2
9	4	0	0	0	3

* Same dog. ** One dog *Anaplasma* spp. antibody (+) and *B. conradae* PCRs (+); another dog *Ehrlichia* spp. antibody (+) and *B. conradae* PCRs (+). ^1^ Antibody detection. ^2^ 18s rRNA RT-PCR and COX1 PCR detection.

## Data Availability

The data presented in this study is contained within the article.
